# Preparing for Future Adversities: Lessons From the COVID-19 Pandemic in Australia for Promoting Relational Resilience in Families

**DOI:** 10.3389/fpsyt.2021.717811

**Published:** 2021-08-04

**Authors:** Ebony J. Biden, Christopher J. Greenwood, Jacqui A. Macdonald, Elizabeth A. Spry, Primrose Letcher, Delyse Hutchinson, George J. Youssef, Jennifer E. McIntosh, Craig A. Olsson

**Affiliations:** ^1^Centre for Social and Early Emotional Development, School of Psychology, Faculty of Health, Deakin University, Geelong, VIC, Australia; ^2^Centre for Adolescent Health, Murdoch Children's Research Institute, Parkville, VIC, Australia; ^3^Department of Paediatrics, Royal Children's Hospital, University of Melbourne, Parkville, VIC, Australia; ^4^National Drug and Alcohol Research Centre, Faculty of Medicine, University of New South Wales, Sydney, NSW, Australia; ^5^The Bouverie Centre, School of Psychology & Public Health, La Trobe University, Melbourne, VIC, Australia

**Keywords:** social support, relationships, family, postpartum, preconception, young adult, resilience, prospective

## Abstract

**Background:** The COVID-19 pandemic has placed considerable pressure on families, testing the quality of relationships and the strength of social support within and beyond the family network. However, little is known about the pre-pandemic factors that predict family relational resilience and social functioning during times of natural disaster or global crisis. Here we use data from one of Australia's longest running studies of social and emotional development to examine the nature and timing of possible relational and social support intervention aimed at preparing families for future adversities.

**Methods:** Data were from the Australian Temperament Project Generation 3 (ATPG3) Study, a population representative three generation cohort study of families established in 1983. A subset of Generation 2 parents completed a COVID-19 specific survey in May-September 2020 (502 parents of 871 children; 60% mothers; 37–38 years). These participants had completed the Quality of Relationships Inventory to assess social support during young adulthood, at 23–24 years (2006) and 27–28 years (2010), before next generation conception. Participants had also completed the Maternity Social Support Scale 1 year postpartum for each child born across the ATPG3 assessment period (2012–2019). In 2020, during the height of the Australian lockdowns, participants rated the quality of their relationships with their partners, children and broader family and friends, in addition to social support within and extended beyond their family.

**Results:** Pre-pandemic partner support was associated with partner relationship quality during the pandemic (β = 0.22). Pre-pandemic support from friends was associated with relationship quality with other family and friends during the pandemic (β = 0.12 – 0.18). Pre-pandemic support (from partner, family and friends) was consistently associated with social support within families during the pandemic (β = 0.11 – 0.21). Pre-pandemic support from friends was also associated with family support extended to others within their local community during the pandemic (β = 0.12 – 0.13).

**Conclusions:** Strengthening supportive relationships during major life transitions, prior to the start of family life and in early parenthood, may have long-term and intergenerational benefits years into the future for both families and communities. This may promote resilience during future crises and other more normative stressful life events.

## Introduction

Global health crises, and large scale disasters more generally, cause families significant stress (1–3). Reciprocal social support within quality family relationships plays a key role in coping with adversity, and in protecting individual and collective health and well-being during times of heightened stress, such as that imposed by the current coronavirus pandemic (2–4). During times of crisis, family and community resilience is in part shaped by social processes that reflect prior experiences in close relationships (5, 6). However, little is known about the developmental antecedents of relational resilience and social support within the family, as well as support extended beyond the family, particularly during global disasters, given that long-term prospective studies that measure these interrelated domains remain rare.

Despite the SARS-CoV-2 infection rate being relatively low compared to other countries (7), Australians have experienced regular lockdowns, some spanning several months at a time, as well as severe mobility restrictions, physical distancing measures, and school and work closures, designed to restrict the spread of the virus (8). Evidence to date indicates that the pandemic has had significant social, psychological, economic, and cultural ramifications (9), including negative impacts on family functioning and mental health and well-being, particularly for parents (10–15). Many families have also experienced enduring stress and insecurity from the widespread loss of employment and income, and remain uncertain about what the future holds for themselves and the next generation (4), signalling the likely ongoing nature of stress for parents and families.

Quality family relationships are a well-documented protective factor capable of buffering negative outcomes in the face of adversity (2–4). The benefits of supportive relationships are observable in a wide range of life domains, and across all stages of the lifecourse (5, 6, 16–22). Quality relationships provide individuals with a critical reserve of coping resources with which to respond to stress and its effects (23). These include internal resources such as a safe haven to express vulnerability, support in regulating emotions or assistance in reappraising stressors (4, 6), and/or external coping resources such instrumental or financial support (24).

Social support within families is also a well-documented protective factor with potential to prevent and buffer stress (2–4, 25–27). Under threat, there is a tendency to focus investments and preserve resources by giving and receiving social support within the family network (3, 28, 29). This serves a critical function of maintaining secure and supportive close relationships, and ensures that support will be reciprocated in future acts of social exchange (6). Wider reaching forms of prosocial behaviours, that extend beyond the family, also play an important role in community resilience and social cohesion, especially during a crisis (1, 2). In this way, social support sustains a cycle of “goodwill,” representing an ongoing resource and source of security and well-being (5, 24). The global COVID-19 pandemic is one of the most striking contemporary examples of the importance of social support at the global level, with the fate of those most vulnerable across the world resting on collective social action and cooperation (30–33).

The extent to which family social networks have been able to sustain positive relationships and provide secure reciprocal support within and outside the family network during the COVID-19 pandemic has been variable (4, 9). This variation is likely influenced by a wide range of pre-pandemic relational and social factors (4, 10, 15). However, little is known about these pre-existing factors, which in turn, limits proactive investment in strengthening population level resilience through enhanced relational and social support. Experiences of social support in close relationships, before and after the transition to parenthood, may be particularly important because they establish longer term internal working models of relationships that shape future expectations of support within later family life (5, 6, 25).

Social support on transition to parenthood can profoundly impact the quality of parents' relationships with their partners and children, as well as their capacity to extend support to others (34, 35). Social support during this period is also key to parental mental health and well-being (36, 37), and by consequence, the family environment and next generation offspring social and emotional development (38–40). Although parents often rely on their partners and family members for support (17, 18), relationships with friends are also protective during this time (41, 42). Social support experienced during this typically positive (albeit still stressful) major life transition may be a key factor in the long-term resilience and thriving of families (6).

Experiences of social support in even earlier transitional periods, in particular those before becoming a parent, may also play a role in shaping relational resilience in the context of life adversity. Young adulthood is a watershed period of change where developmental tasks undergo consolidation in preparation for a wide range of challenges related to education, employment, community engagement, partner selection, and the roles and responsibilities of adult life (43). Young adults rely heavily on close and supportive relationships to meet these challenges (43). Peers are particularly crucial in this period (17), while family support remains an important resource (43). Experiences of social support before next generation conception (preconception), across the 20's, may also establish expectations of relationships and social support in future family life.

Prospective cohort studies that have measured social health and development before and during the COVID-19 pandemic are uniquely placed to investigate the antecedents of relational resilience and social support within the family, as well as extended support beyond the family. Studies that have followed cohorts before becoming parents, and then into the parenting years, are further placed to provide insights into the long-term development of risk and resilience pathways. Here, we access unique prospective data from a 38-year old population-based cohort study. We examine the extent to which social support up to 14 years before the pandemic, in young adulthood (preconception) and in early parenthood (postpartum), are associated with relationship quality and social support within and beyond the family during the COVID-19 pandemic in Australia. We disaggregate associations by: (1) types of parent relationship (with partner, with children, and with broader family and friends); (2) types of social support (within family, within community, and globally); and (3) sources of pre-pandemic social support (from partner, family, and friends).

## Methods

### Participants

The Australian Temperament Project Generation 3 (ATPG3) Study is a 38-year population-based cohort that has three waves of Generation 3 perinatal data and 15 waves of Generation 1 (grandparent; G1) and Generation 2 (parent; G2) preconception data. The study commenced in 1983 as a population representative survey of the social and emotional health of 2,443 infants (4–8 months) and their parents (The Australian Temperament Project; ATP). Families were followed up via mail surveys approximately every 2 years until G2 age 19–20 years, and every 4 years thereafter (44). Between 2012 and 2018, the cohort was screened biannually for pregnancies, with all identified expecting parents invited into the third-generation (ATPG3) study. Assessments occurred at three time points across the perinatal period by phone: at 32 weeks pregnancy, and 8 weeks and 1 year postpartum. Across this period, 1,167 Generation 3 (G3) offspring born to 703 ATP parents were recruited into the ATPG3 cohort.

From May to September 2020 during the height of the Australian COVID-19 lockdowns, all G2 study members participating in the ATPG3 study with one or more children were invited to complete a COVID-19 specific online survey module assessing the impacts of the pandemic. A total of 516 G2 parents (60% female; 37–38 years) of 891 G3 offspring completed the survey. Those who participated in the COVID survey were representative of all ATPG3 participants on baseline variables (G2 sex, infant difficult temperament, and behaviour problems, as well as G1 education and country of birth). We excluded participants not living in Australia at the time (*n* = 14), resulting in a final sample size of 502 ATPG3 parents (60% female) of 871 G3 offspring in the current study.

The ATPG3 Study protocols were approved by the Royal Children's Hospital Human Research Ethics Committee. Prior waves were approved by human research ethics committees at the University of Melbourne, the Australian Institute of Family Studies and/or the Royal Children's Hospital, Melbourne. All data have been collected and stored in the REDCap database (45).

### Measures

#### COVID-19 Outcomes

##### Relationship Quality During the Pandemic (2020)

Parents reported on the quality of their relationships over the past 2 weeks with their partner (one item; “How would you rate the quality of your relationship with your partner?”), their children (one item; “How would you rate the quality of your relationship with your child?”), and their broader family and friends (one item; “How would you rate the quality of your relationships with other family and/or friends?”). For all items, responses were given on a 5-point scale with 1 = *very poor*, 2 = *not so good*, 3 = *mixed*, 4 = *quite good* and 5 = *very good*, so that for each of the three outcomes, higher scores indicated better relationship quality. Partner relationship quality for parents not in a relationship (*n* = 19) was coded as missing. When parents had more than one child participating in the study, scores for each child were averaged to reflect the overall quality of parents' relationships with their children. The average correlation between children was *r* = 0.47.

##### Social Support During the Pandemic (2020)

Parents reported on the level of within family support over the past 2 weeks (one item; “To what extent have members of your family supported each other when upset or struggling with any aspect of the outbreak?”), family support provided to others within their local community (one item; “To what extent have you, or others in your household, provided practical, emotional and/or financial support to other people in your community struggling with the outbreak?”), and family support provided to others globally (one item; “To what extent have you, or others in your household, provided practical, emotional and/or financial support to people in other countries struggling with the outbreak?”). For all items, responses were given on a 5-point scale with 1 = *almost never*, 2 = *rarely*, 3 = *sometimes*, 4 = *often* and 5 = *almost always*, so that for each of the three outcomes, higher scores indicated greater support.

#### Pre-pandemic Exposures

##### Postpartum Social Support (2012–2019)

The Maternity Social Support Scale (MSSS) (46) was used to prospectively assess social and emotional support at 1 year postpartum, across the 8 year ATPG3 perinatal assessment period (2012–2019, up to 8 years pre-pandemic). The MSSS is a self-report survey consisting of six items assessing perceived social support. Items assessed support from family (one item; “My family is always there for me”), friends (one item; “I have good friends who support me”), and partner (four items; e.g., “My husband/wife/partner helps me a lot,” and “I feel loved by my husband/wife/partner”). For all items, responses were given on a 5-point scale with 1 = *never*, 2 = *rarely*, 3 = *some of the time*, 4 = *most of the time* and 5 = *always*. Scores were calculated for total, partner, family, and friend social support, so that higher scores indicated greater social support. For parents with multiple children participating in the study, we selected scores from the most recent postpartum assessment. The MSSS has shown good reliability and predictive utility postpartum (46, 47).

##### Preconception Social Support (2006–2010)

The Quality of Relationships Inventory (QRI) (48) was used to prospectively assess preconception social support on two occasions during young adulthood, at 23–24 years (wave 14, 2006, 14 years pre-pandemic) and 27–28 years (wave 15, 2010, 10 years pre-pandemic). The QRI is a self-report measure, with the social support scale consisting of items assessing relationship-based perceived support. At each wave, three items (“You can count on them to listen to you,” “You can turn to them for advice,” and “You can count on them for help with a problem”) were completed with respect to both family (parent/s) and friends. For all items, responses were given on a 5-point scale with 1 = *never*, 2 = *rarely*, 3 = *sometimes*, 4 = *often* and 5 = *always*. For total, family, and friend social support, scores were derived such that higher scores indicated greater social support. An average score was taken across ages 23–24 and 27–28 years. The QRI has shown sound internal consistency, temporal stability, construct validity, and predictive utility in young adult populations (49).

#### Potential Confounders

Distal (preconception and pre-pandemic) confounders theorised to be associated with social development and related outcomes were selected. These were identified as factors up to the time of exposure assessment (G2 age 28 years) and included participant family background characteristics of G1 country of birth (either parent born outside of Australia), low G1 education (< secondary school), and G1 separation or divorce. G2 participant characteristics were also controlled for including sex, mental health (self-reported average level of depression [Short Moods and Feelings Questionnaire (50)] and anxiety symptoms [age 13–14 years, adapted from the Revised Behaviour Problems Checklist Short Form (51); age 15-18 years, Revised Children's Manifest Anxiety Scale (52)]), as well as anti-social behaviour (self-reported average frequency of eight antisocial behaviours, e.g., damaged things in a public place, stolen something or been in physical fights with others), during adolescence (13–18 years, waves 10–12, 1996–2000).

### Statistical Analysis

All data were analysed using Stata 16 (53). Generalised estimating equations with an exchangeable working correlation were used to estimate linear regressions with multivariate COVID-19 outcomes of relationship quality and social support. Models estimated the relationships between source of social support (i.e., total, partner, family, and friends) at each pre-pandemic exposure period (i.e., postpartum and preconception) and the COVID-19 outcomes. Specifically, in separate models each COVID-19 outcome (three indicators included simultaneously) were regressed onto each measure of pre-pandemic social support. To determine associations with the specific indicators of each outcome, models included an interaction between pre-pandemic social support and a variable denoting the outcome indicator. Models were adjusted for all potential confounding factors. Additionally, for models examining postpartum social support, we further accounted for the time between postpartum and COVID-19 assessment waves. For pandemic relationship quality, we also conducted sensitivity analyses excluding participants without a partner (*n* = 19).

Missing data in the analysis sample ranged from 1 to 27%. Multiple imputation was used to address potential biases due to missing data. All variables were included in the imputation model. Twenty complete data sets were generated, based on a multivariate normal model (54). Binary variables were imputed as continuous variables, then back transformed with adaptive rounding following imputation (55). Results were pooled across the 20 imputed datasets using Rubin's rules to obtain regression estimates (56). Following imputation, all COVID-19 outcomes and pre-pandemic exposure variables were standardised (*z* scores), so that effect sizes (β) are interpreted as a change in standard deviation units of pandemic relationship quality or social support for every standard deviation increase in pre-pandemic social support.

## Results

### Descriptives

A descriptive summary of the unstandardised COVID-19 outcomes, pre-pandemic exposures, and potential confounding variables are detailed in Table 1, alongside the percent of missing data. Overall, parents rated the quality of their relationships during the pandemic as “quite good” (with partners *M* = 4.15, *SD* = 0.96; with children *M* = 4.45, *SD* = 0.66; and with other family and friends *M* = 3.93, *SD* = 0.83). Parents reported that family members “often” supported each other within the family (*M* = 4.11, *SD* = 1.04), which attenuated to “sometimes” supporting others within their local community (*M* = 2.92, *SD* = 1.18) and “rarely” supporting people globally during the pandemic (*M* = 1.66, *SD* = 1.10). Parents reported that they felt supported “most of the time” prior to the pandemic (1 year postpartum: *M* = 4.42, *SD* = 0.45), and that they felt supported “often” well-before the pandemic (preconception: *M* = 4.42, *SD* = 0.50).

**Table 1:** Descriptive statistics for COVID-19 outcomes, pre-pandemic exposures and potential confounding factors in the unimputed data (*n* = 502 parents of 871 children).

	***M***	***SD***	**95% CI**	**% missing**
**Relationship quality during the pandemic^a^**				
Partner	4.15	0.96	(4.06, 4.23)	4%
Children	4.45	0.66	(4.39, 4.51)	1%
Other family and friends	3.93	0.83	(3.85, 4.00)	1%
**Social support during the pandemic^a^**				
Within family	4.11	1.04	(4.02, 4.20)	2%
Within community	2.92	1.18	(2.82, 3.03)	1%
Globally	1.66	1.10	(1.56, 1.76)	1%
**Pre-pandemic postpartum social support^b^**				
Total	4.42	0.45	(4.37, 4.47)	27%
Partner	4.43	0.51	(4.38, 4.48)	27%
Family	4.53	0.76	(4.45, 4.61)	27%
Friends	4.28	0.88	(4.19, 4.37)	27%
**Pre-pandemic preconception social support** ^c^				
Total	4.42	0.50	(4.38, 4.47)	7%
Family	4.52	0.65	(4.46, 4.58)	8%
Friends	4.32	0.63	(4.27, 4.38)	7%
**Potential confounding factors**				
Adolescent mental health	−0.02	0.77	(−0.09, 0.05)	14%
Adolescent anti-social behaviour	0.19	0.25	(0.17, 0.22)	8%
	***n*** **(cases)**	**%**	**95% CI**	**% missing**
G1 country of birth outside Australia	142	29%	(25, 33%)	3%
G1 low education	111	22%	(19, 26%)	0%
G1 separation	146	29%	(26, 34%)	1%

a *Pandemic = 2020*.

b *Postpartum = 1 year postpartum, 2012–2019*.

c *Preconception = young adulthood, 2006–2010*.

### Pre-pandemic Social Support and Associations With Relationship Quality and Social Support During the Pandemic

Associations between pre-pandemic social support (total and disaggregated by partner, family and friend sources) and each parent relationship during the pandemic (with partner, children, and other family and friends) are presented in Table 2. Following adjustment for potential confounders, total pre-pandemic social support postpartum was associated with the quality of other family and friend relationships during the pandemic [β = 0.17 (95% CI 0.07, 0.28)]. When examined by source of pre-pandemic social support, the strongest associations were observed between social support from partner pre-pandemic (postpartum) and partner relationship quality during the pandemic [β = 0.22 (95% CI 0.11, 0.34)]. Social support from friends pre-pandemic, both postpartum [β = 0.18 (95% CI 0.08, 0.28)] and preconception [β = 0.12 (95% CI 0.03, 0.22)], was also associated with the quality of other family and friend relationships during the pandemic. There was negligible evidence that social support from family pre-pandemic, both postpartum and preconception, was related to the quality of any relationships during the pandemic. Analyses excluding participants without a partner were consistent with the above results and are presented in Supplementary Table 1.

**Table 2:** Adjusted associations between pre-pandemic social support and relationship quality during the COVID-19 pandemic.

**Pre-pandemic social support**	**β**	**95% CI**	***p***	**β**	**95% CI**	***p***	**β**	**95% CI**	***p***
	**Relationship quality during the pandemic^a^**								
	**Partner**			**Children**			**Other family and friends**		
**Postpartum^b^**									
Total	0.08	(−0.03, 0.20)	0.155	0.08	(−0.04, 0.19)	0.196	0.17	(0.07, 0.28)	0.001
Partner	0.22	(0.11, 0.34)	<0.001	0.03	(−0.08, 0.13)	0.632	0.08	(−0.02, 0.19)	0.118
Family	0.01	(−0.10, 0.12)	0.807	0.04	(−0.07, 0.15)	0.467	0.08	(−0.03, 0.18)	0.151
Friends	0.00	(−0.11, 0.10)	0.939	0.08	(−0.04, 0.19)	0.191	0.18	(0.08, 0.28)	<0.001
**Preconception^c^**									
Total	0.02	(−0.08, 0.11)	0.712	0.06	(−0.04, 0.15)	0.225	0.07	(−0.03, 0.16)	0.168
Family	−0.02	(−0.11, 0.07)	0.694	0.03	(−0.06, 0.13)	0.491	−0.02	(−0.11, 0.07)	0.715
Friends	0.05	(−0.05, 0.14)	0.312	0.06	(−0.03, 0.16)	0.200	0.12	(0.03, 0.22)	0.009

*Each row represents a discrete regression*.

a *Pandemic = 2020*.

b *Postpartum = 1 year postpartum, 2012–2019*.

c *Preconception = young adulthood, 2006–2010*.

Associations between pre-pandemic social support (total and disaggregated by partner, family and friend sources) and social support during the pandemic (within family, within community and globally) are presented in Table 3. Following adjustment of potential confounders, the strongest associations were observed between social support within families and total social support pre-pandemic, both postpartum [β = 0.21 (95% CI 0.10, 0.32)] and preconception [β = 0.16 (95% CI 0.07, 0.25)]. These results were consistent when examined across all sources of pre-pandemic support [β = 0.11 – 0.15]. Family support provision to others within their local community was also associated with pre-pandemic social support from friends, with similar effect sizes across postpartum [β = 0.13 (95% CI 0.03, 0.24)] and preconception [β = 0.12 (95% CI 0.03, 0.22)].

**Table 3:** Adjusted associations between pre-pandemic social support and social support during the COVID-19 pandemic.

**Pre-pandemic social support**	**β**	**95% CI**	***p***	**β**	**95% CI**	***p***	**β**	**95% CI**	***p***
	**Social support during the pandemic^a^**								
	**Within family**			**Within community**			**Globally**		
**Postpartum^b^**									
Total	0.21	(0.10, 0.32)	<0.001	0.08	(−0.03, 0.19)	0.157	0.07	(−0.03, 0.18)	0.167
Partner	0.14	(0.03, 0.25)	0.012	0.00	(−0.11, 0.10)	0.957	0.04	(−0.06, 0.15)	0.433
Family	0.14	(0.04, 0.25)	0.009	0.00	(−0.11, 0.11)	0.979	0.06	(−0.05, 0.18)	0.291
Friends	0.15	(0.04, 0.27)	0.008	0.13	(0.03, 0.24)	0.014	0.04	(−0.06, 0.15)	0.411
**Preconception^c^**									
Total	0.16	(0.07, 0.25)	0.001	0.09	(−0.01, 0.18)	0.073	0.06	(−0.03, 0.16)	0.184
Family	0.11	(0.02, 0.20)	0.019	0.01	(−0.08, 0.10)	0.804	0.03	(−0.06, 0.13)	0.484
Friends	0.14	(0.05, 0.23)	0.003	0.12	(0.03, 0.22)	0.010	0.07	(−0.03, 0.16)	0.165

*Each row represents a discrete regression*.

a *Pandemic = 2020*.

b *Postpartum = 1 year postpartum, 2012–2019*.

c *Preconception = young adulthood, 2006–2010*.

## Discussion

Using prospective data, we examined the extent to which experiences of social support in the preconception and postpartum periods, up to 14 years before the pandemic, were later associated with the quality of parents' close relationships and social support levels within and beyond the family during the COVID-19 pandemic in Australia. We found a pattern of within-source associations whereby relationship quality with partners and other family and friends during the pandemic was associated with a history of social support from partners (postpartum) and friends (postpartum and preconception), respectively. Additionally, within family support during the pandemic was consistently associated with a history of pre-pandemic social support from all sources, during both postpartum and preconception periods. Finally, extending support to the community during the pandemic was associated with pre-pandemic social support from friends, during both postpartum and preconception periods. Our results show that higher pre-pandemic levels of social support, during key transitional periods, are related to better relational functioning during the pandemic. Promoting supportive relationships both within and external to the family environment during young adulthood through to early parenthood may be an important intervention target for future public health efforts, in order to strengthen pro-social protective pathways within and across generations in preparation for future global crises.

Notably, in regard to relationship quality during the pandemic, our results suggest a pattern of continuity in partner and friend social support from young adulthood and early parenthood up to 14 years before the pandemic, into the period of the COVID-19 pandemic. Specifically, those with better supportive relationships tended to maintain these levels over time and as a result may be more resilient during periods of stress. Relationships with significant close others, such as partners and friends, may represent interdependent interactions within which social support can be reciprocally exchanged over time (20). Our findings suggest that foundational social support within these relationships may be involved in the maintenance and promotion of the quality of these connections under times of heightened stress. Partners and close friends may develop the skills, knowledge and motivation to provide responsive and sensitive reciprocal support to each other, preserving their social bonds (6). Although individual partners and friends may change over long periods of time, the patterns, dynamics and instrumental nature of each type of relationship may tend be established earlier in the lifecourse and remain relatively stable (5). Our findings suggest a clear continuity within source over time, whereby intervention on social support may need to also be source specific to obtain the most benefits to future relationship quality.

In contrast to the continuity of associations with partner and friend support, similar patterns were not observed for social support from family. Pre-pandemic social support from family, both postpartum and preconception, did not appear to be related to the quality of relationships during the pandemic, with partner, children or other family and friends. One explanation may be that this reflects the normative shift away from identifying with the family network as young adults build their sense of autonomy (17, 43). Additionally, this may reflect a tendency of parents to build a support network of “chosen family,” preferencing more emotionally meaningful and fulfilling connections rather than traditionally conceptualising the family, to create positive environments to raise their children in (17, 57). Alternatively, this finding may have a methodological explanation. Items examining family support may have been answered by balancing a range of support experiences within the family (both positive and negative, and with many different sources in mind), which may have weakened the predictive utility of the measure.

Similarly, parents' relationship quality with their children during the pandemic, did not appear to be related to parents' pre-pandemic levels of social support, neither in the postpartum or preconception period, nor from any particular source of support. The smaller effect sizes observed for associations between parents' histories of social support and the quality of their relationships with their children during the pandemic may reflect the asymmetrical nature of the parent-child relationship, such that the parent is generally the provider of support. The quality of this unique relationship may be driven by more deeply embedded processes not assessed here such as attachment orientations (5), or factors more proximal to the pandemic including experiences of home schooling and/or increased quality time spent together. Alternatively, this may again have a methodological explanation, given that relationship quality with children was assessed using a single summary item. Despite our finding that parental histories of social support were not related to the quality of their relationships with their children when under stress, social support remains an important resource for parents in creating a supportive family environment in which to raise their children (34, 35).

When examining associations between social support within the family and extending beyond the family during the pandemic, we found that all sources of both postpartum and preconception social support were associated with family members providing support to each other during the pandemic. Additionally, both postpartum and preconception social support from friends was associated with providing support into the community. This supports previous research on the principle of reciprocity, that receiving and perceiving support tends to increase the likelihood of providing support to others in the future (58, 59). Our findings demonstrate that this process can be observed over more than a decade, and even under times of significant adversity when people are most in need of support. Parents' default support responses during periods of crises may be those that have been learnt through modelling or previous behavioural exchanges (5, 6, 25, 38). As such, support from partner and family prior to the pandemic may reflect internal working models of expectations of providing support to family members, and assist in creating supportive family environments in the future. Similarly, support from friends during young adulthood and early parenthood may represent the pathway of learning to engage in social support, both receiving and providing it, with those beyond the family unit into the community. Our findings point to the importance of promoting non-insular, compassionate, peer and community support that extends beyond families during major life transitions, as it may have longitudinal and intergenerational benefits.

However, we did not find similar evidence of associations between parental histories of social support and family support globally during the pandemic. Our results suggest that this process of longitudinal reciprocity may be source specific, and only extend to those whom individuals are interdependent on; who they feel socially connected to, rely on, and to whom they can see the impact of their support. This may represent the phenomena of tightening ones' social network when under threat, in order to focus resources on protecting kin (3, 28, 29). Despite the global scale of the COVID-19 crisis, the limited generalisation of family support globally, may also be a product of pandemic-related restrictions placed on travel and connections overseas. Moreover, as we have primarily found source specific associations over time, provision of support to those in other countries may be better predicted by more specific pre-pandemic factors such as family or friends living outside Australia, engagement in overseas travel or aid, or the extent to which people feel connected with global humanity (31).

Effect sizes were strongest for exposures in the more proximal postpartum period, as might be expected for factors closer in time (60). The smaller effects we observed are of public health interest, given that young adult assessment occurred 14 years prior to assessment during the pandemic and that relationship quality and social support are multi-determined (60, 61). Our findings highlight the likelihood of a multitude of accumulating and cascading influences on relational and social development, which have consequences for all domains and stages of the lifecourse (60, 61). Social support is only one of many resilience factors which might be important during a global health crisis (4).

### Strengths and Limitations

A key strength of this study is its multi-wave longitudinal design, with social support measured prospectively more than a decade prior to the pandemic. This allowed us to identify sources of social support during specific transitional periods across the early adult lifecourse that might be important in shaping future families' relational and social adaptive functioning during large scale crises. Some limitations should also be considered. Although the sample is a population-based cohort, participants were predominantly white and Australian born (representing the demographics of the state of Victoria, Australia in 1983). As with all longitudinal studies, some bias due to differential attrition is also likely, despite participants broadly representing those eligible on baseline characteristics. Future research should investigate these associations in more diverse populations and vulnerable groups such as culturally and linguistically diverse communities and families of children with additional needs.

Levels of missing data were low in the achieved sample and were addressed using multiple imputation. We also adjusted for key demographic variables, however, as with all observational studies, the potential influence of unmeasured confounding remains. Our social support and relationship quality measures were brief, to reduce participant burden, and future research may examine more nuanced types of support. Additionally, we did not investigate gender differences in associations due to low power. This is an important line of inquiry for future research given that men and women have been differentially impacted by the COVID-19 pandemic (62), and that gendered socialisation may affect the capacity to seek, receive and reciprocate support (63–65). Furthermore, although we were able to capture the relational and social climate within families during the early stages of the pandemic during which extensive and strict lockdowns were implemented, it will be important to examine the role of parents' social support histories in protecting family socio-emotional well-being in the longer term.

### Implications and Translation

Findings from this study, if replicated, raise important questions about how we might prepare families to cope with future natural disasters. Findings confirm the importance of accruing supportive relationships across the early adult years for later resilience within “the family of procreation” under times of crisis (2, 4). If causal, our findings suggest that interventions aimed at promoting supportive relationships could be impactful from as early as young adulthood through to becoming a parent and beyond. The focus of these interventions could be on helping young adults develop secure and meaningful connections with family and peers in order to facilitate greater availability and perceptions of support during this crucial stage of development and into the future. Strengthening social support may not only promote individual resilience, but also influence the support extended to others within families and communities. Thus, strengthening supportive relationships creates the foundation for resilient communities, which will be an important factor in addressing calls for greater pandemic preparedness (32, 33, 66).

The current coronavirus pandemic has also demonstrated the need to encourage global pro-sociality and cooperation to support those most vulnerable in our communities, and simultaneously reduce risk to the global collective (30–33). This principle is likely to apply in future global disasters such as those resulting from climate change, and our results suggest that strengthening individual social support during major life transitions may promote pro-social support to the wider community in times of crisis. Building this relational resilience could occur in a range of settings including socio-emotional learning and relationship programs in schools and higher education institutes as well as clubs and youth programs in communities. Moreover, social support during the early postpartum period, particularly from partner and friends, represents an additional later potential point of intervention to foster family resilience.

## Conclusions

Parents' recent and distal histories of social support up to 14 years before the pandemic were associated with subsequent relationship quality and social support within and beyond the family during the COVID-19 pandemic. Strengthening a diverse range of supportive relationships during young adulthood prior to the start of family life and in early parenthood may have long-term and intergenerational benefits within families and communities. Findings from this study have the potential to inform lifecourse approaches to preventing vulnerability and promoting resilience and coping in the context of public health emergencies. These processes may translate across other life stressors and more normative major stressful life events. Social support is not only important in coping with stress, but also in helping individuals, families, and communities thrive in their everyday lives (6).

## Data Availability Statement

The datasets presented in this article are not readily available because ethics approvals do not permit the data to be made publicly available due to limitations of participant consent and concerns regarding potential re-identifiability. Requests to access the datasets should be directed to https://lifecourse.melbournechildrens.com/data-access/ where submissions can be made using our institutional data access protocols.

## Ethics Statement

The studies involving human participants were reviewed and approved by the Royal Children's Hospital Human Research Ethics Committee. The participants provided their written informed consent to participate in this study.

## Author Contributions

EJB, CJG, JAM, EAS, PL, and CAO: conceptualization, design, and drafting of the manuscript. EJB and CJG: statistical analyses. All authors interpretation of data, critical revision, and final acceptance of manuscript.

## Author Disclaimer

The views expressed in this paper are those of the authors and may not reflect those of their organisational affiliations, nor of other collaborating individuals or organisations.

## Conflict of Interest

The authors declare that the research was conducted in the absence of any commercial or financial relationships that could be construed as a potential conflict of interest.

## Publisher's Note

All claims expressed in this article are solely those of the authors and do not necessarily represent those of their affiliated organizations, or those of the publisher, the editors and the reviewers. Any product that may be evaluated in this article, or claim that may be made by its manufacturer, is not guaranteed or endorsed by the publisher.
