# Supplementary material for: Preparing for Future Adversities: Lessons From the COVID-19 Pandemic in Australia for Promoting Relational Resilience in Families
Source: Front Psychiatry. 2021 Aug 4;12:717811. doi: 10.3389/fpsyt.2021.717811 (PMC8371315; doi:10.3389/fpsyt.2021.717811)
Supplement: Supplementary file 1 [file Table_1.DOCX]

| Supplementary Table 1. Adjusted associations between pre-pandemic social support and relationship quality during the COVID-19 pandemic excluding participants without a partner (*n* = 19) | | | | | | | | | |
| --- | --- | --- | --- | --- | --- | --- | --- | --- | --- |
| Pre-pandemic social support | *β* | 95% CI | *p* | *β* | 95% CI | *p* | *β* | 95% CI | *p* |
|  | Relationship quality during the pandemic ^a^ | | | | | | | | |
|  | Partner | | | Children | | | Other family and friends | | |
| Postpartum ^b^ |  |  |  |  |  |  |  |  |  |
| Total | 0.08 | (-0.03, 0.19) | 0.155 | 0.08 | (-0.04, 0.19) | 0.186 | 0.18 | (0.08, 0.29) | 0.001 |
| Partner | 0.23 | (0.11, 0.35) | <0.001 | 0.03 | (-0.08, 0.14) | 0.596 | 0.09 | (-0.02, 0.20) | 0.108 |
| Family | 0.01 | (-0.09, 0.12) | 0.796 | 0.05 | (-0.06, 0.17) | 0.353 | 0.09 | (-0.02, 0.20) | 0.101 |
| Friends | 0.00 | (-0.12, 0.11) | 0.946 | 0.07 | (-0.05, 0.18) | 0.252 | 0.19 | (0.08, 0.29) | <0.001 |
| Preconception ^c^ |  |  |  |  |  |  |  |  |  |
| Total | 0.01 | (-0.08, 0.11) | 0.790 | 0.04 | (-0.05, 0.14) | 0.375 | 0.06 | (-0.03, 0.16) | 0.200 |
| Family | -0.02 | (-0.11, 0.07) | 0.654 | 0.03 | (-0.07, 0.13) | 0.563 | -0.02 | (-0.12, 0.07) | 0.658 |
| Friends | 0.04 | (-0.05, 0.14) | 0.352 | 0.04 | (-0.05, 0.14) | 0.381 | 0.12 | (0.03, 0.22) | 0.011 |
| Note: Each row represents a discrete regression.  ^a^ Pandemic = 2020; ^b^ Postpartum = one year postpartum, 2012-2019; ^c^ Preconception = young adulthood, 2006-2010. | | | | | | | | | |

Supplementary Material
